# URJC GB dataset: Community-based seed bank of Mediterranean high-mountain and semi-arid plant species at Universidad Rey Juan Carlos (Spain)

**DOI:** 10.3897/phytokeys.35.6746

**Published:** 2014-03-25

**Authors:** Patricia Alonso, José María Iriondo

**Affiliations:** 1 Área de Biodiversidad y Conservación, Departamento de Biología y Geología, Escuela Superior de Ciencias Experimentales y Tecnología, Universidad Rey Juan Carlos, Tulipán s/n, 28933, Móstoles, Spain

**Keywords:** Accessions, germplasm bank, gypsum, high-mountain, Madrid, seeds, seedbank, semi-arid, Spain, Universidad Rey Juan Carlos, wild species

## Abstract

The Germplasm Bank of Universidad Rey Juan Carlos was created in 2008 and currently holds 235 accessions and 96 species. This bank focuses on the conservation of wild-plant communities and aims to conserve *ex situ* a representative sample of the plant biodiversity present in a habitat, emphasizing priority ecosystems identified by the Habitats Directive. It is also used to store plant material for research and teaching purposes. The collection consists of three subcollections, two representative of typical habitats in the center of the Iberian Peninsula: high-mountain pastures (psicroxerophylous pastures) and semi-arid habitats (gypsophylic steppes), and a third representative of the genus *Lupinus*. The high-mountain subcollection currently holds 153 accessions (63 species), the semi-arid subcollection has 76 accessions (29 species,) and the *Lupinus* subcollection has 6 accessions (4 species). All accessions are stored in a freezer at -18 °C in Kilner jars with silica gel. The Germplasm Bank of Universidad Rey Juan Carlos follows a quality control protocol which describes the workflow performed with seeds from seed collection to storage. All collectors are members of research groups with great experience in species identification. Herbarium specimens associated with seed accessions are preserved and 63% of the records have been georreferenced with GPS and radio points. The dataset provides unique information concerning the location of populations of plant species that form part of the psicroxerophylous pastures and gypsophylic steppes of Central Spain as well as populations of genus *Lupinus* in the Iberian Peninsula. It also provides relevant information concerning mean seed weight and seed germination values under specific incubation conditions. This dataset has already been used by researchers of the Area of Biodiversity and Conservation of URJC as a source of information for the design and implementation of experimental designs in these plant communities. Since they are all active subcollections in continuous growth, data is updated regularly every six months and the latest version can be accessed through the GBIF data portal at http://www.gbif.es:8080/ipt/resource.do?r=germoplasma-urjc. This paper describes the URJC Germplasm Bank and its associated dataset with the aim of disseminating the dataset and explaining how it was derived.

## Data published through GBIF

http://www.gbif.es:8080/ipt/resource.do?r=germoplasma-urjc

## Introduction

### The URJC Germplasm Bank

**Figure 1.**
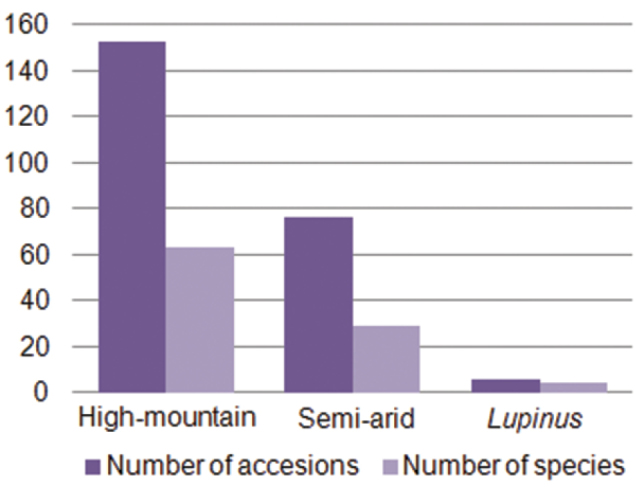
Number of accessions and species in the different subcollections of the URJC GB.

The URJC Germplasm Bank (URJC GB) was created in 2008 and focuses on the conservation of wild plant communities. Until now, germplasm banks have conserved plant biodiversity and have promoted the use of plant genetic resources at the species, subspecies or variety level, conserving threatened taxa or taxa with some important traits. In these cases, the aim is to conserve the species or variety regardless of its origin or natural habitat. However, as the most appropriate way to conserve a biological entity is within the ecosystem that it naturally forms part of (Gómez-Campo 1985, Prance 1997), the concept of seed banks can be reconsidered, at least for wild species. In this context, the objective of this germplasm bank is to conserve *ex situ* a representative sample of the entire plant biodiversity characteristic of a habitat with special emphasis on habitats defined as priority habitats by the Habitats Directive. The URJC GB is used to conserve seeds for research projects and teaching purposes. It currently holds 235 accessions and 96 species. Figure 1 shows the number of accessions and species in each subcollection. Thus, there are 153 accessions and 63 species in the high-mountain subcollection, 76 accessions and 29 species in the semi-arid subcollection and 6 accessions and 4 species in the *Lupinus* subcollection.

### The URJC GB dataset

Creating and managing a germplasm bank not only entails keeping the seed material under proper storage conditions, but also ensuring the correct administration of all the related data. After all, seed accessions with no associated information are virtually useless from a conservation and research perspective. Thus, it is essential for seed banks to obtain reliable data and record it in a database accessible to potential users. In addition to passport data on the location, time and other features of seed collection, additional data need to be gathered throughout the different activities involved in seed preservation, including seed processing, testing and storage. These data must be accurately compiled and efficiently managed to ensure the accuracy, consistency and wider relevance of the dataset. This information is necessary for both the management of the seed bank and for conservation and research studies on the species included in the seed bank.

Most global and national efforts in the dissemination of seed bank datasets are related to plant species of agricultural value. In this sense, it is worthy to mention the Germplasm Resources Information Network (GRIN) of the United States Department of Agriculture (http://www.ars-grin.gov/), and the EURISCO catalogue of the European Cooperative Programme for Plant Genetic Resources (ECPGR) (http://eurisco.ecpgr.org/), which provides information about *ex situ* plant collections maintained in Europe. Furthermore, many datasets are available at the genebank, national, subregional and regional levels (see Bettencourt (2011) for a detailed account). Concerning wild plant species seed banks, dataset availability is scarce. For instance,of the 13 wild plant germplasm banks in Spain, only BG JBB (Germplasm Bank of Botanic Garden of Barcelona) and URJC GB have currently published their dataset through GBIF, although there are ongoing efforts to make all this information available through the Spanish Network of Seed banks (REDBAG, http://www.redbag.es/). A good example of a dataset of this type of seed banks is provided by the Data Warehouse of the Millenium Seed bank Partnership coordinated by Royal Botanic Gardens, Kew (http://herbaria.plants.ox.ac.uk/bol/msbp).

**Figure 2.**
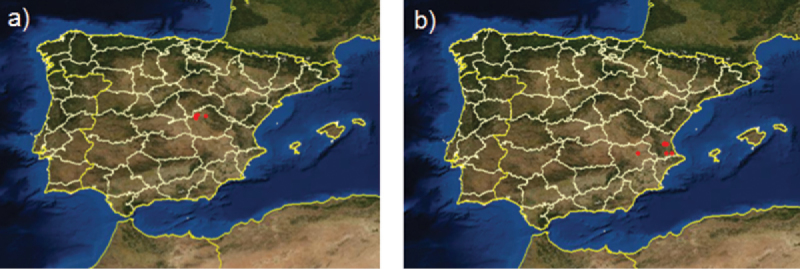
Distribution map of the two species of URJC GB dataset endemic to Spain: **a** *Helianthemum marifolium* ssp. *conquense* **b** *Lupinus mariae-josephae* (Source: http://www.anthos.es).

This dataset provides unique information on the occurrence of plant species in two characteristic habitats of Spain that are currently protected by the Habitats Directive of the European Union (Council Directive 92/43/EEC). Some of the species recorded in the dataset are endemic (i.e., *Helianthemum marifolium* ssp. *conquense*, *Lupinus mariae-josephae*) (Figure 2) or have special threat status. The information contained in this dataset has already been used in several research articles published in scientific journals as a source of information on chorology, seed germination and phenology. Furthermore, the storage of seeds in the seed bank allows experiments to be replicated in these studies if necessary.

## Dataset description

**Object name:** Darwin Core Archive Banco de Germoplasma de la Universidad Rey Juan Carlos

**Character encoding:** UTF-8

**Format name:** Darwin Core Archive format

**Format version:** 1.0

**Distribution:** http://www.gbif.es:8080/ipt/archive.do?r=germoplasma-urjc

**Publication date of data:** 2013-12-05

**Language:** English

**Licenses of use:** This database “URJC GB dataset” is made available under the Open Data Commons Attribution License: http://www.opendatacommons.org/licenses/by/1.0/.

## Project description

**Project title:** URJC GB dataset: Community-based seed bank of Mediterranean high-mountain and semi-arid plant species at Universidad Rey Juan Carlos.

**Personnel:** José María Iriondo (Principal Investigator) and Patricia Alonso.

**Funding:** This project is financed by LIMITES (CGL2009-07229) and AdAptA (CGL2012-33528) research projects of the Spanish Ministry of Science and Innovation and Remedinal-2 project of the Autonomous Community of Madrid.

**Study area description:** The study area includes high-mountain Mediterranean systems and semi-arid Mediterranean ecosystems of Peninsular Spain. It also comprises ruderal habitats linked to the occurrence of *Lupinus* species. The sampled high-mountain Mediterranean systems are psycroxerophyllous pastures in the Sierra de Guadarrama mountain range located between 1550 and 2438 m elevation. The sampled semi-arid Mediterranean systems correspond to plant communities of gypsum steppes in the river Tajo valley located between 489 and 939 m elevation. Finally, the ruderal habitats of *Lupinus* spp. are scattered in Central Spain between 440 and 743 m elevation. Further details concerning the climate, geologic substrate and vegetation of these habitats are provided in the spatial coverage section. The study area is currently represented by the provinces of Ávila, Cuenca, Guadalajara, Madrid, Salamanca, Segovia, Valencia and Zaragoza in Spain.

**Figure 3.**
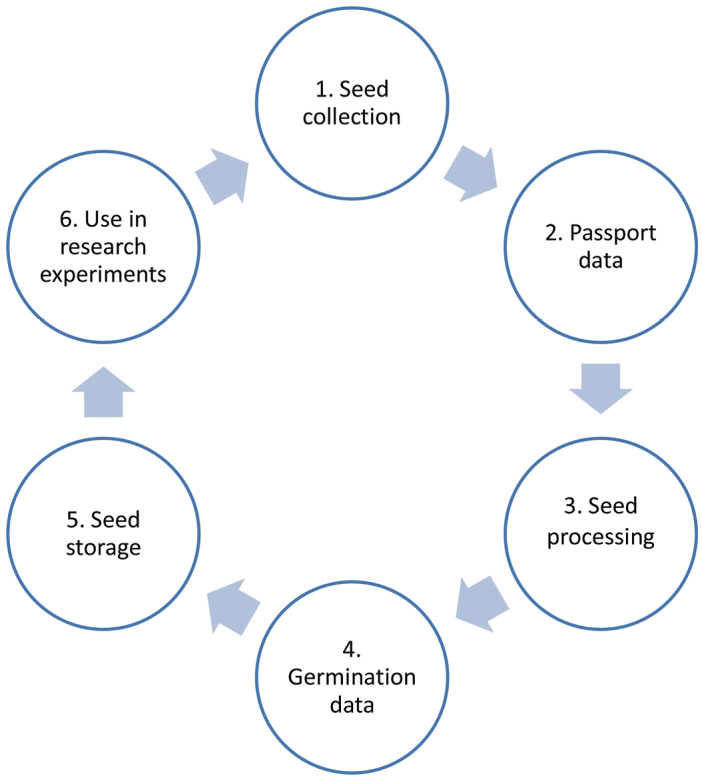
Basic elements of the project design. **1** Seed collection in the target plant communities **2** Gathering of passport data on the geographical, physical and biotic features of the locality of seed collection **3** Seed processing to prepare seed accessions **4** Initial germination experiments to obtain seed germinability **5** Seed storage in cold-dry conditions **6** Use in research experiments.

**Design description:** The Germplasm Bank of Universidad Rey Juan Carlos is a long-term research infrastructure project which aims to collect and conserve seeds of plant species that are found in the ecosystems where most of the research of the Biodiversity and Conservation Area of URJC takes place. In addition to conserving seed accessions of the most representative species of these plant communities, this project aims to gather and manage data describing the geographic, physical and biotic characteristics of the associated habitat of each accession and the morphological and physiological traits of the seeds. Collected and preserved seeds can also be used in research experiments focused on comparing the performance of germplasm across a spatial or temporal range. Figure 3 summarizes the basic elements of the project design. Further details on the seed collection and preservation process are given in the Sampling Methods section.

## Taxonomic coverage

### General taxonomic coverage description

The URJC GB holds the seeds of vascular plants from specific habitats. The high-mountain subcollection is composed of communities of siliceous psicroxerophilous pastures dominated by *Festuca curvifolia* and rich in hemicryptophytes and chamaephytes. The semi-arid subcollection is composed of gypsophytes characteristic of semi-arid environments dominated by *Cistaceae*, *Asteraceae* and *Labiatae*. Finally, the *Lupinus* subcollection is composed of seeds from different species in this genus collected in different regions in Spain.

**Figure 4.**
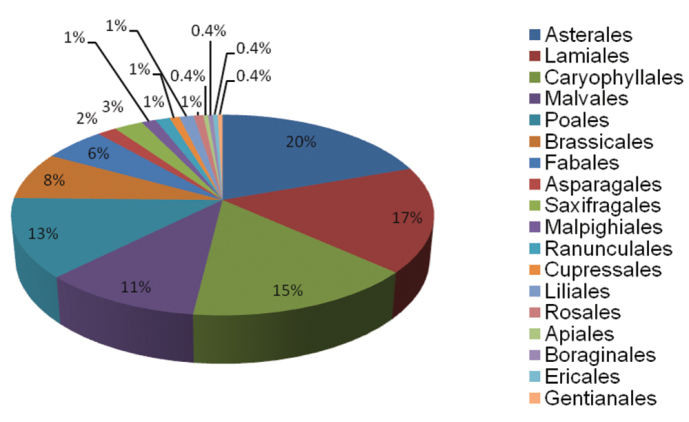
Taxonomic coverage (percentage per order) of URJC GB.

**Figure 5.**
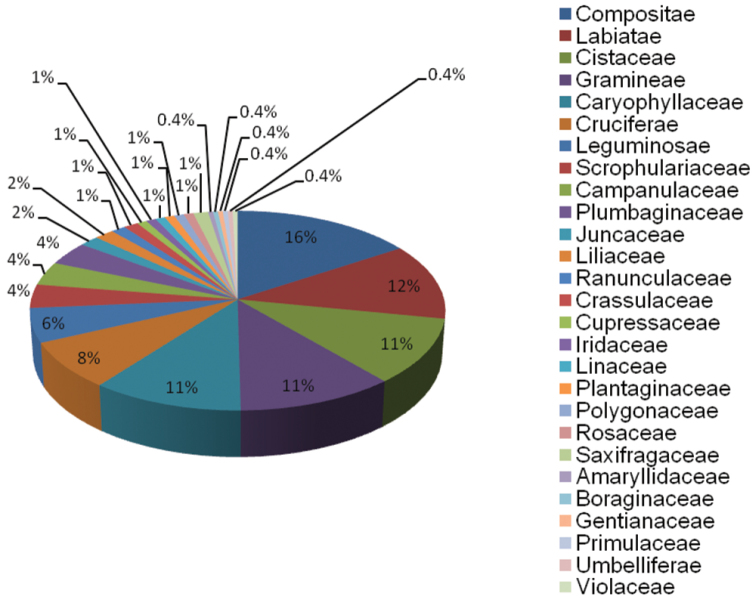
Taxonomic coverage (percentage per family) of URJC GB.

Two phyla are represented in the URJC GB: Magnoliophyta (198; 99%) and Pinophyta (2; 1%). Of the three classes found, the most representative is Magnoliopsida (167; 84%), followed by Liliopsida (31; 15%) and Pinopsida (2; 1%). 18 orders are represented in the Germplasm Bank, and those with the highest number of accessions are Asterales, Lamiales, Poales, Malvales, Caryophyllales, Brassicales and Fabales (Figure 4). Of the 27 families in the collection, those with the highest representation are Compositae, Labiatae, Cistaceae, Gramineae, Caryophyllaceae, Cruciferae and Leguminosae (Figure 5).

### Taxonomic ranks

**Kingdom:** Plantae

**Phylum:** Magnoliophyta, Pinophyta

**Class:** Liliopsida, Magnoliposida, Pinopsida

**Order:** Apiales, Asparagales, Asterales, Boraginales, Brassicales, Caryophyllales, Cupressales, Ericales, Fabales, Gentianales, Lamiales, Liliales, Malpighiales, Malvales, Poales, Ranunculales, Rosales, Saxifragales

**Family:** Compositae, Labiatae, Cistaceae, Gramineae, Caryophyllaceae, Cruciferae, Leguminosae, Scrophulariaceae, Campanulaceae, Plumbaginaceae, Juncaceae, Liliaceae, Ranunculaceae, Crassulaceae, Cupressaceae, Iridaceae, Linaceae, Plantaginaceae, Polygonaceae, Rosaceae, Saxifragaceae, Amaryllidaceae, Boraginaceae, Gentianaceae, Primulaceae, Umbelliferae, Violaceae.

## Spatial coverage

### High-mountain subcollection

**Figure 6.**
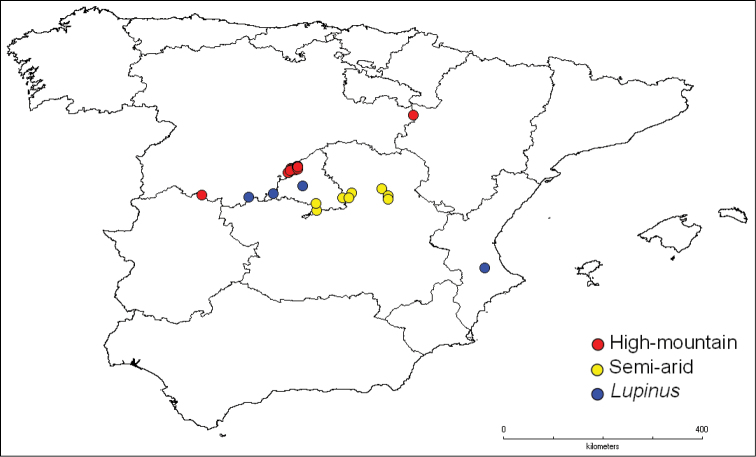
Geographical distribution of the germplasm subcollections in Peninsular Spain.

The seeds in this subcollection are from different areas along the altitudinal gradient of the Sierra de Guadarrama between the provinces of Madrid, Segovia and Ávila in Central Spain (Figure 6). This mountain range follows a southwest-northeast orientation and is approximately 80 Km long. It forms part of the Sistema Central, which delimits the hydrographic basins of the Tajo and Duero Rivers. With regard to its lithologic composition, it is dominated by plutonic and metamorphic siliceous rocks like granite, gneiss, slate and quartzite.

Peñalara Natural Park extends over 768 hectares and is located in the municipality of Rascafría (Madrid). The altitude of the Park ranges from 1640 m to 2428 m (Peñalara Peak), and the highest peaks of the Sierra de Guadarrama are found here. Habitats vary along the altitudinal gradient: from lower to higher elevations, *Pinus sylvestris* woods, shrublands dominated by *Cytisus oromediterraneus* and *Juniperus communis* subsp. *alpina*, and different summit pastures dominated by *Festuca curvifolia*. There are also azonal habitats that are dependent on geomorphology and hydrological and edaphic conditions, such as screes, rocky areas and fens (Vielva et al. 2004).

Climate conditions as well as its relative geographic isolation have made Peñalara Peak a biogeographic island where species characteristic of colder areas and higher latitudes have found refuge, forming the southern limit of their distribution. These species are difficult to find in other parts of Central Spain and include species such as *Senecio boissieri*, *Coincya orophila*, *Erysimum penyalarense* and *Armeria caespitosa* (Fernández-González 1987).

Mean monthly temperatures on the highest peaks range between 0 °C in the winter months and 6 °C in the summer months. Minimum and maximum temperatures range between -18 °C and 26 °C, respectively. Annual precipitation is around 1300 mm, with a large proportion in the form of snow (Peñalara Natural Park, pers. com.).

The altitude of this area confers climatic conditions characteristic of high mountains. This is shown by the high contrast in daily temperatures, strong winds, extreme minimum temperatures, the accumulation of snow during long periods, high insolation in summer accompanied by seasonal drought and high UV radiation (Giménez-Benavides et al. 2007, García-Romero and Muñoz-Jiménez 2010).

This habitat (number 6160) is included in the Habitats Directive of the European Union (Council Directive 92/43/EEC) due to its singularity and reduced area (Rivas Martínez 1963, European Community 1992, Giménez-Benavides 2006).

### Semi-arid subcollection

The seeds in this subcollection are from different areas along the altitudinal gradient in the Tajo valley. These communities occur at 700–900 m elevation in the gypsum steppes in the southeastern area of the Madrid Autonomous Region and in the northeastern area of the Castilla - La Mancha Autonomous Region (Guadalajara and Cuenca) (Figure 6). These gysophyte communities are characteristic of dry, poorly developed gypsum soils, which often have a lichen crust. They are generally open communities dominated by chamaephytes and small bushes, sometimes accompanied by *Stipa tenacissima*. Among the gypsophilous shrubs, gypsophyte communities of annual ephemeral plants can also be found (Martín-Herrero et al. 2003).

These communities generally occur in the mesomediterranean floor in semi-arid or dry ombroclimates. They rarely penetrate the supramediterranean floor or occur in subhumid ombroclimates, where conditions are no longer favourable for their development, and they are substituted by other communities from base-rich soils (Martín-Herrero et al. 2003). Mean annual precipitation ranges between 429 and 596 mm, mean annual temperature between 12 and 13.7 °C and mean minimum temperature in the coldest month between 0.8 and 2.2 °C (Elías, Ruiz 1981, in Ferrandis et al. 2005).

The different texture and composition of gypsum soils influence the floristic composition of this vegetation. These peculiar communities are exclusive to Iberian steppes and have a high number of plants, including several endemics. This, together with their adaptation to a substrate that is very selective for other plant communities, makes them of singular interest. They are considered a “priority habitat” (number 1520) according to Council Directive 92/43/EEC (European Community 1992, Martín-Herrero et al. 2003).

### Lupinus subcollection

The seeds in this subcollection are from different locations in Spain: Madrid, Valencia y Ávila (Figure 6). In this case, they are not from a specific ecosystem, and samples are collected from any region within the territory.

### Coordinates

38°58'58,8"S and 40°51'3,6"N Latitude; 5°43'4,8"W and 0°31'1,2"E Longitude.

## Temporal coverage

**Figure 7.**
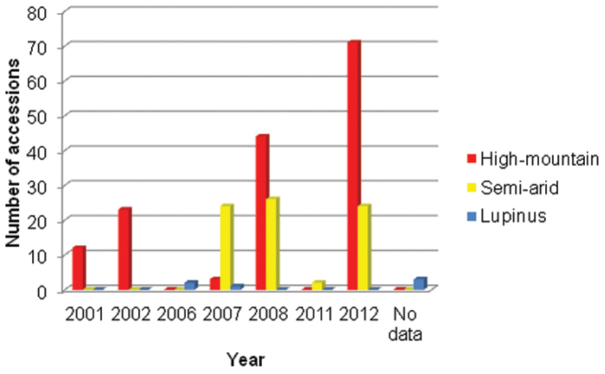
Temporal coverage in the different subcollections of the URJC GB.

2001 – 2012. The earliest collection event dates back to 2001 and the latest to 2012. The highest number of accessions was collected in 2012, 2008 and 2006 for the High-mountain subcollection, the Semi-arid subcollection and the *Lupinus* subcollection, respectively (Figure 7). Three accessions in the *Lupinus* subcollection lack year data. There are significant variations in the number of seeds collected between years due to the number and type of research projects active at the institution at a given time. Thus, seed collecting expeditions are carried out according to the experimental design of each project involved. It is worth noting that all subcollections are active and in continuous growth. Therefore, temporal coverage will be regularly updated.

## Sampling methods

### Study extent description

**Figure 8.**
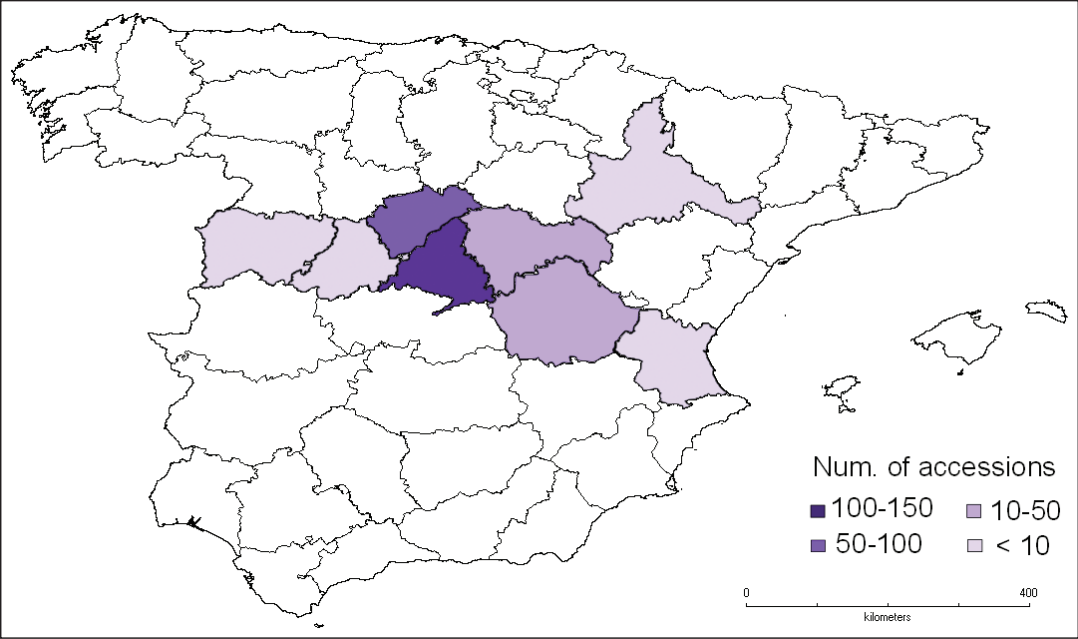
Geographical distribution of accessions in Peninsular Spain.

The Sierra de Guadarrama and gypsophylic steppes of Central Spain are the most significant areas represented in URJC GB. Seeds have been collected from 8 provinces in Spain: Ávila, Cuenca, Guadalajara, Madrid, Salamanca, Segovia, Valencia and Zaragoza (Figure 8). 84% of the accessions come from Madrid and Segovia.

### Sampling description

In the collection phase, seed samples were collected from several different localities to cover the distribution range of the communities in the study area and thereby obtain genetic material from the different species with the potential to adapt to different local conditions.

To plan seed collection, it was essential to determine the timing of seed dispersal. In this sense, the literature related to the phenology of the collected species was consulted. Other morphological indicators of the timing of natural dispersal were also used. These included tissue hardness, changes in color in seeds and fruits, dryness of pods and capsules, among others.

The physical quality of the seeds was evaluated at the time of collection to avoid collecting specimens that were infected or clearly unviable. We aimed to collect between 3000 and 5000 seeds per accession to guarantee that there would be enough material for germination assays, long-term conservation and, if necessary, propagation (Gold et al. 2004). In order not to compromise the viability of the population *in situ*, no more than 20% of the seeds present at the time of collection were sampled (Gold et al. 2004).

Once samples were collected for each species, they were placed in paper bags and labeled with the name of the species, the site where they were collected, altitude, site coordinates, the date of collection and the name of the collector. This information constituted the passport data for each seed accession.

### Quality control

The URJC GB has a protocol which describes the seed processing methods for collection, processing in the laboratory, germination assays, dessication, recording accessions, labeling, scanning and freezing. Seed collectors are professors and researchers with great knowledge of the flora characteristic of each ecosystem. They are all members of research groups that have worked in this field for years and, therefore, have great experience in the phenology and identification of the species. Herbarium specimens associated with the seed accessions are preserved at the Department of Biology and Geology of URJC or at the Herbarium of the Royal Botanic Garden of Madrid. 63% of the records are georreferenced with GPS and radio points. When this information is not available, the geographic coordinates of the site and the extension of the municipality are used as a surrogate of this measure. Since the subcollections are active and continuously growing, data are updated regularly every six months.

### Description of steps

**Figure 9.**
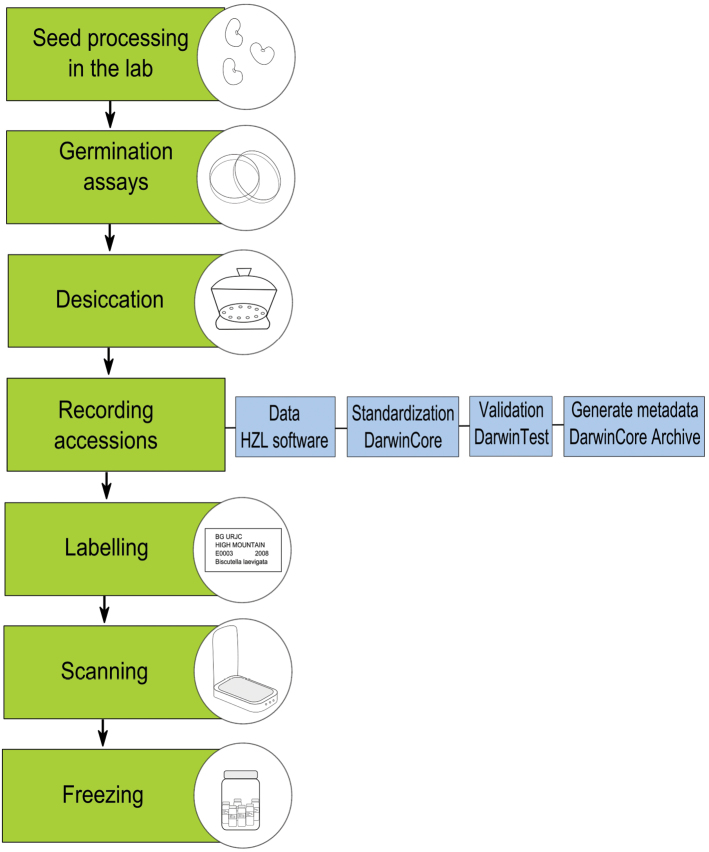
URJC Germplasm Bank workflow.

The different steps of the process are summarized in Figure 9 and are described as follows:

**Seed processing in the laboratory:** The first treatment consisted of cleaning the seeds. Sieves with stainless steel meshes of different sizes (mesh holes between 2 mm–0.5 cm) were used to separate as much undesired material as possible (soil, stones, small leaves, stems, flowers, etc.) from the seed sample. The separated seeds were then introduced in tubes identified with the passport data (species name, date, site, altitude and collector) and stored in a cupboard at room temperature under ambient moisture conditions.

**Germination assays:** Germination assays were carried out with 100 seeds of each accession. Four replicates of 25 seeds were placed in Petri dishes on two pieces of filter paper. Distilled water was added until the surface was wet, and the dishes were placed in a germination chamber (Selecta Hotcold GL, Barcelona, Spain). Accessions from the high-mountain subcollection were incubated at 15 °C with a 16/8 hour light/dark photoperiod (Giménez-Benavides et al. 2005), while accessions from the semi-arid subcollection were incubated at 20 °C with the same photoperiod (Pérez-García and Durán 1989, Escudero et al. 1997, Herranz et al. 2002). Assays were monitored every 2–3 days for a minimum period of one month, and the assay was considered to have finished when no germination was observed for four subsequent censuses (8-day period). Germinated seeds were counted and removed in each census.

**Desiccation:** Silica gel (SiO_2_) was used to desiccate the samples. As desiccation rates increase with the amount of gel used, the base of the desiccator was filled to a 1:1 proportion with the seeds. The seeds, which were separated by accessions in the Petri dishes, were placed uncovered on the metal plate in the desiccator. They were then placed in a cool place to dry for at least two months.

**Recording accessions:** Accessions were added to the database of the URJC GB using Herbar Zoorbar Ligero (HZL) software. Each accession was assigned a reference number allocated consecutively. The information contained in the record of each accession is: dataset, institution, catalogue number, scientific name, family, genus, specific epithet, taxon rank, infraspecific epithet, scientific name authorship, collection code, subcollection, number of mother plants, number of tubes (in which the accession is stored), number of seeds, seed weight, community name, country, country code, state province, municipality, locality, decimal latitude, decimal longitude, geodetic datum, coordinate uncertainty in meters, elevation, collector, number of jar (in which the accession is stored), number of drawer (in which the accession is stored), collection date, sample acquisition date, % germination and observations. All data are standardized by DarwinCore 1.2 and validated by Darwin Test. We generated metadata with DarwinCore Archive in order to publish data in GBIF IPT.

**Labelling:** After registering the accession in the database, seed accessions were labelled for proper identification.

**Scanning:** Approximately 50 seeds were uniformly distributed avoiding contact between them and scanned. A resolution of 300 ppp was used, except in the case of very small seeds when a 600 ppp resolution was used.

**Freezing:** All of the subcollections in the URJC GB are base collections; i.e. they are stored for long periods of time and are only used in regeneration processes. Storage is carried out under optimal conditions to maximize seed viability. The procedure followed at the URJC GB was to store seeds in glass tubes in Kilner jars with silica gel. These jars were sealed hermetically and placed in a freezer at -18 °C.
